# Case Report: A Case Report and Literature Review on Severe Bullous Skin Reaction Induced by anti-PD-1 Immunotherapy in a Cervical Cancer Patient

**DOI:** 10.3389/fphar.2021.707967

**Published:** 2021-08-24

**Authors:** Xiang Li, Li-Xin Qu, Yu-Mei Ren, Chang Hu

**Affiliations:** ^1^ The Fifth Department of Oncology, Jinshazhou Hospital of Guangzhou University of Chinese Medicine, Guangzhou, China; ^2^ The Second Clinical Medical College, Henan University of Chinese Medicine, Zhengzhou, China; ^3^ Pediatric Ward, Henan Province Hospital of TCM, Zhengzhou, China; ^4^ Department of Oncology, Fuda Cancer Hospital Guangzhou, Guangzhou, China

**Keywords:** severe bullous skin reactions, literature review, case report, toxic epidermal necrolysis, cervical cancer, PD-1

## Abstract

**Background:** Anti-programmed cell death protein 1 (PD-1) has been successfully used in carcinomas treatment. However, it causes significant adverse effects (AEs), including cutaneous reactions, particularly the life-threatening severe bullous skin reactions (SBSR) and toxic epidermal necrolysis (TEN).

**Case summary:** Herein, we described for the first time a case report of SBSR induced by anti-PD-1 therapy in a cervical cancer patient. In addition, we revised existing literature on anti-PD-1 induced cutaneous reactions. We reported a cervical cancer patient who was treated with four successive cycles of Sintilimab and Toripalimab injections and developed systemic rashes, bullae, and epidermal desquamation, which worsened and led to infection, eventually causing death after being unresponsive to aggressive treatments.

**Conclusion:** Anti-PD-1 antibodies commonly cause skin toxicity effects, some of which may be deadly. Therefore, healthcare providers should observe early symptoms and administer proper treatment to prevent aggravation of symptoms.

## Introduction

Cervical cancer is the fourth most common cancer in women (Colombo et al., 2012). Its main treatment consists of platinum-based chemotherapy, with limited therapeutic outcomes and severe side effects (Greer et al., 2010; Pfaendler and Tewari, 2016). Since the introduction of programmed death 1 (PD-1) protein monoclonal antibodies, they have shown outstanding clinical efficacy in multiple cancer types, including advanced cervical cancer (Martínez and Del Campo, 2017; Wang and Li, 2019). In this regard, Pembrolizumab was used for advanced cervical cancer in the KEYNOTE-028 clinical study, demonstrating the efficacy of PD-1/PD-L1 inhibitors in advanced cervical cancer (Frenel et al., 2017). PD-1 monoclonal antibodies have been shown to potentiate T lymphocytes cytotoxic activity against tumor cells and control tumor growth (Pedoeem et al., 2014; Tumeh et al., 2014). Most patients tolerated anti-PD-1 therapy, whereas some presented toxic and side effects (Champiat et al., 2016). Major anti-PD-1 associated adverse effects (AEs) included skin toxicity, endocrine reaction, gastrointestinal reaction, hepatitis, and renal dysfunction (Hofmann et al., 2016; Ansell, 2017; Nishijima et al., 2017; O’Kane et al., 2017; Tie et al., 2017; Gubens et al., 2019). The most common AEs involved skin reactions such as lichenoid reaction, eczema, vitiligo, and pruritus (Joseph et al., 2015; Robert et al., 2015a; Weber et al., 2015; Ciccarese et al., 2016; Hwang et al., 2016; Yang et al., 2019). However, the most severe skin reaction observed was toxic epidermal necrolysis (TEN) in three cases of malignant melanoma (Nayar et al., 2016; Vivar et al., 2017; Logan et al., 2020).

## Case Presentations

**FIGURE 1:**
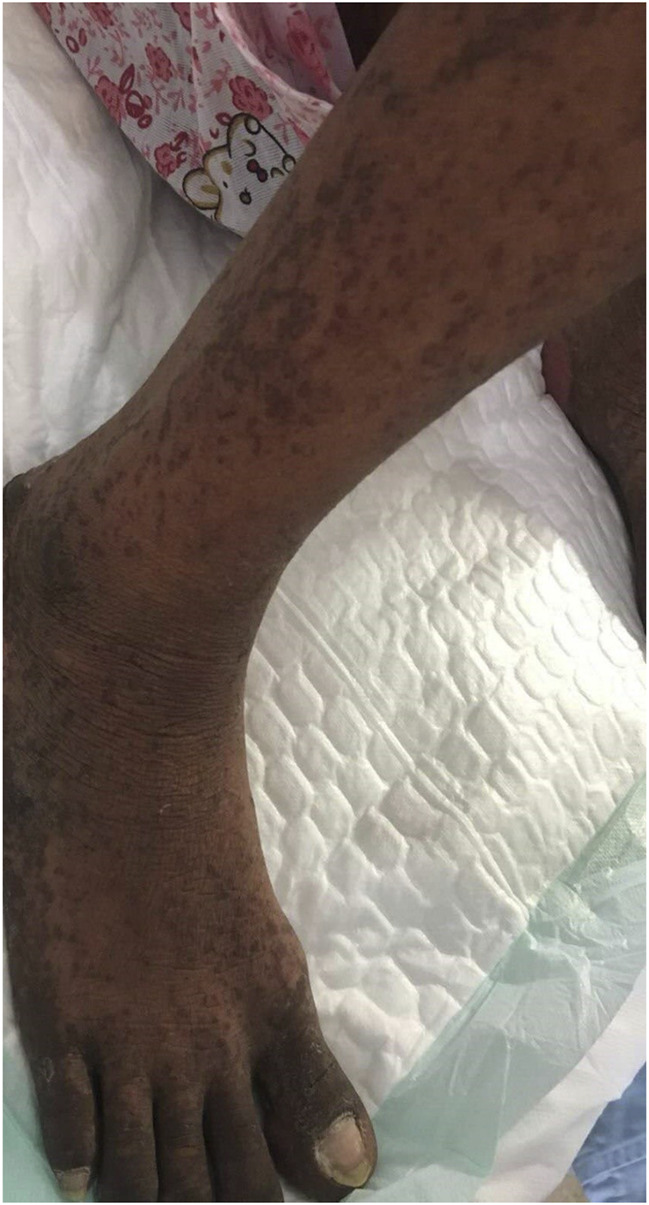
Maculopapular skin rash on legs and feet.

The patient was a 38-year-old Asian female. In June 2019, cervical tumor with invasion of the uterine wall, bladder and rectum walls, and anterior sacral and bilateral inguinal lymphadenopathies was detected by magnetic resonance imaging, which was prescribed because she presented vaginal bleeding. Biopsy pathological results suggested cervical squamous cell carcinoma, FIGO stage IVA. On August 18, 2019, she was intravenously (i. v.) administered 240 mg paclitaxel +90 mg cisplatin chemotherapy, along with 200 mg Sintilimab at 21-days cycle intervals. On September 9, 2019, the patient received a second cycle of the same dose of Sintilimab and chemotherapy. Sintilimab is an innovative monoclonal antibody targeting PD-1, jointly developed by Innovent and Lilly in China, which has been granted marketing approval by the China Food and Drug Administration. The drug was granted orphan drug status by the FDA in April 2020 for the treatment of esophageal cancer. Because of financial issues, Sintilimab was switched to 240 mg Toripalimab in the third cycle on October 1, 2019, for two consecutive weeks per cycle, whereas the chemotherapy regimen remained unaltered. Toripalimab is also an anti-PD-1 monoclonal antibody produced in China. In March 2020, Toripalimab was granted orphan drug status by the US FDA in combination with acytinib for the treatment of mucosal melanoma. A follow-up exam after the third cycle showed progressive disease. In the fourth cycle on October 21, 2019, we modified chemotherapy to 240 mg paclitaxel and 135 mg nedaplatin, combined with 200 mg Sintilimab. Six days after the fourth cycle of treatment, she presented with rashes. Large erythema was observed in many parts of the body, along with some prominent skin areas and pigmentation (Figure 1) and she was given the antihistamine diphenhydramine. The patient further developed shortness of breath and edema of both lower limbs, which was considered a heart failure condition. Cardiotonic, diuretic, and vasodilator agents were then provided. In addition, red blood cell transfusion was given, because her hemoglobin was 61 g/L.

**FIGURE 2:**
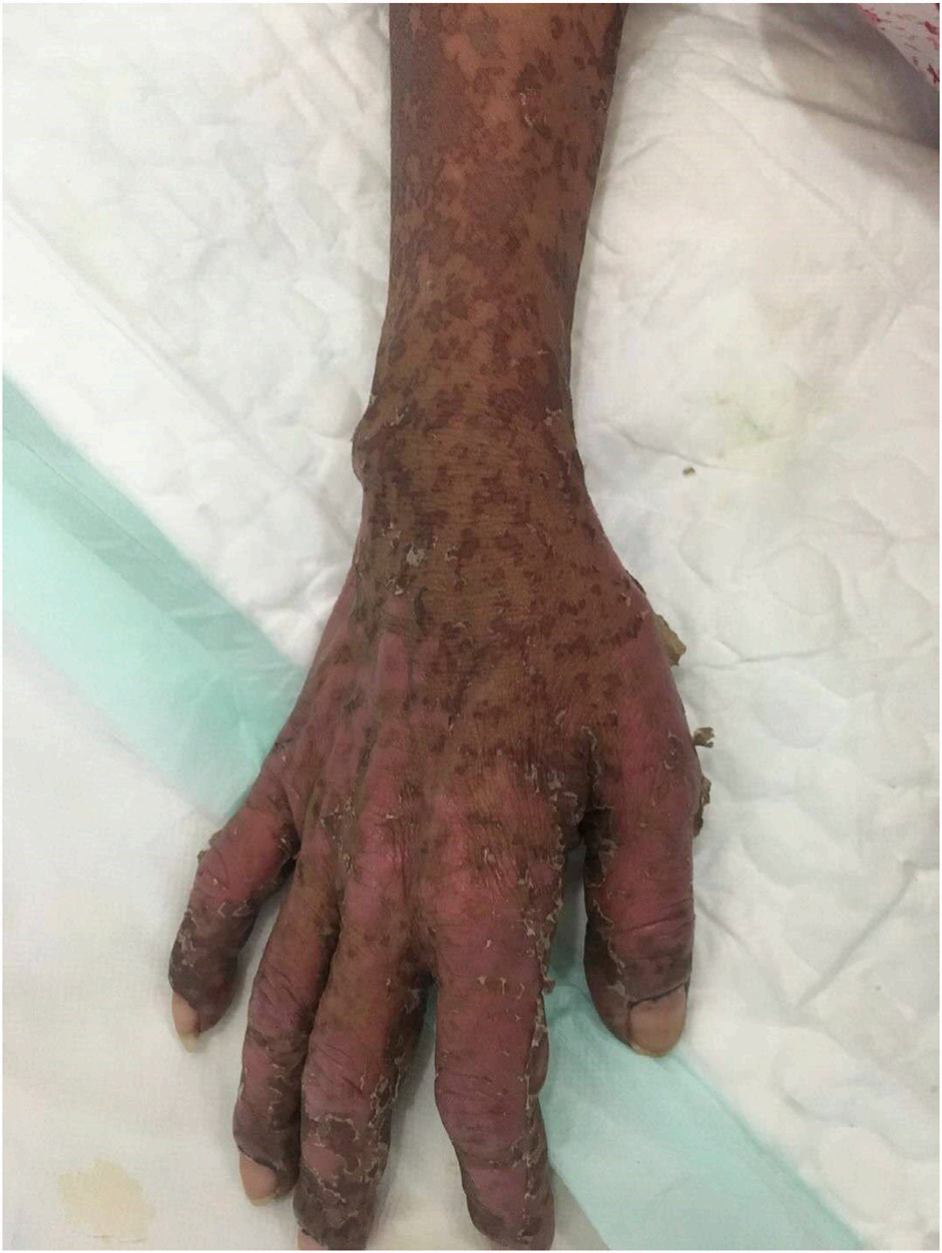
Epidermal exfoliation of upper limbs (BSA less than 10%).

**FIGURE 3:**
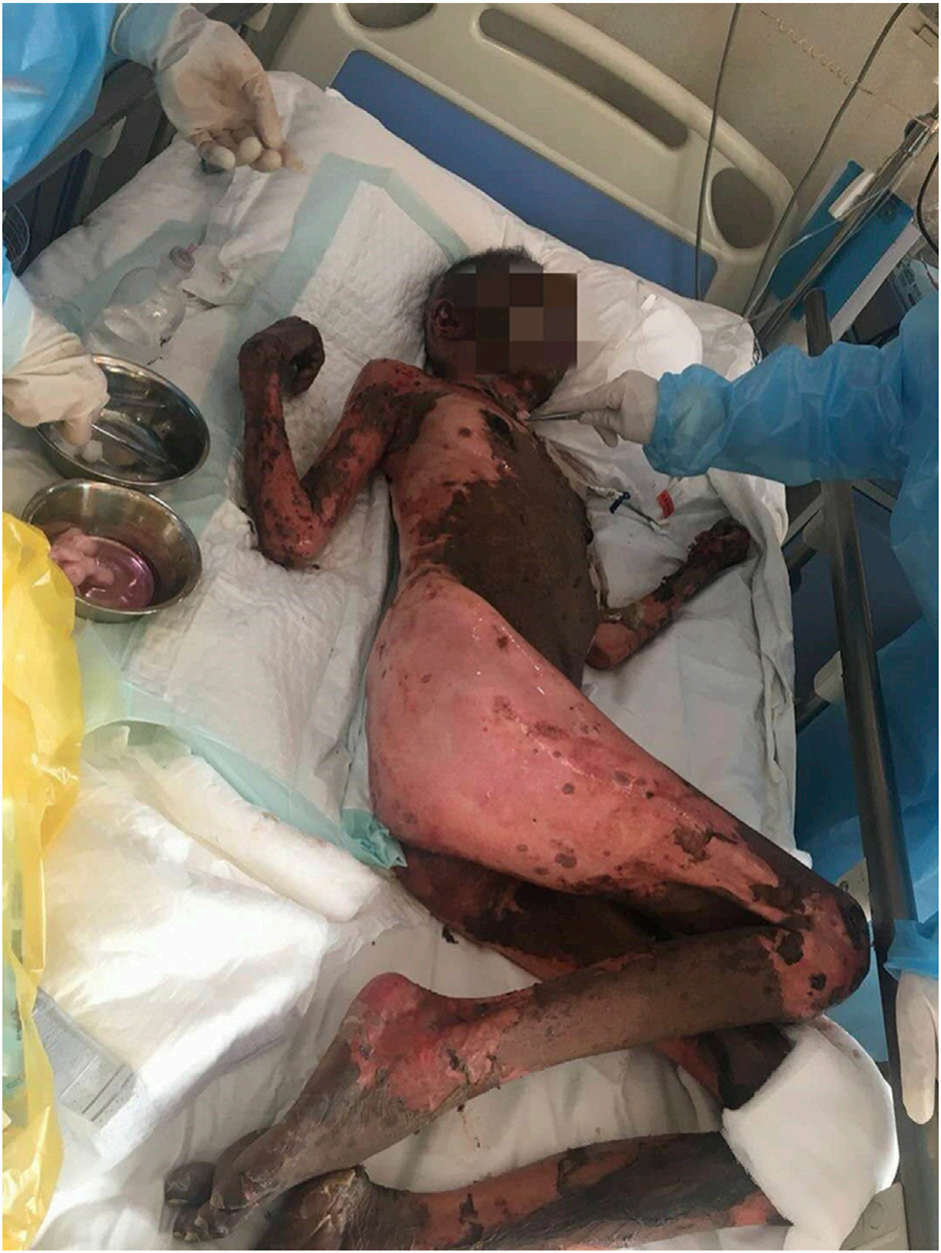
A large exfoliated area of the body (BSA more than 30%).

After antihistamine treatment, she presented with worsening symptoms, with increased erythema, local transparent blisters, and tender epidermal loss of less than 10% of the body surface area (BSA). Furthermore, she developed mild swelling of oropharyngeal mucosa with repeated bleeding and scabbing, resulting in mouth opening and swallowing difficulties (Figure 2), but refused to undergo skin biopsy. Stevens-Johnson syndrome (SJS) was diagnosed by a professor of dermatology, and we started i. v. administration of 80 mg/d methylprednisolone, combined with 0.4 g/kg/d immunoglobulins injection every day. We also improved the skin, mouth, and perineal mucous membranes care. Six days after medication, the epidermal peeling aggravated. Clear blisters locally appeared, presenting apparent perineal skin damage, with skin loss over 30% of BSA **(** Figure 3 **)**. The patient also presented fever, with respective C-reactive protein and neutrophil values of 117.33 mg/L and 89.20%. The skin lesion developed to SBSR, accompanied by infection, which was treated with antibiotics. The epidermal exfoliation and infection continued to worsen, and a disturbance of consciousness was observed. The patient died 33 days after the fourth cycle of medication, probably due to sepsis.

## Discussion

### Cutaneous Adverse Effects of Anti-Programmed Death-1 Therapy

Although docetaxel and paclitaxel share the same pharmaceutical composition, cases of SBSR -causing paclitaxel have not been reported to date (Ohlmann et al., 2007; Kattan et al., 2008; Cohen et al., 2019). The toxicity spectrum of anti-PD-1 is prone to skin-related reactions, excluding the possibility that other drugs used by the patient may cause SBSR, which we believed it was related to anti-PD-1 treatment.

PD-1/PD-L1 pathway inhibits T cell activation, inducing lymphocyte apoptosis, and maintains autoimmune tolerance. In the tumor microenvironment, tumor cells bind to PD-1 on the lymphocyte surface through PD-L1 overexpression to inhibit lymphocytes function, thus escaping the immune surveillance and destruction (Dong and Chen, 2006). PD-1 is an important target in anti-tumor therapy because the aforementioned inhibitory signaling pathways can be blocked, enhancing T cells immune response (Topalian et al., 2012).

In major lung cancer related studies, CheckMate 017 and CheckMate 057 (Borghaei et al., 2015; Brahmer et al., 2015) revealed that anti-PD-1 AEs in lung cancer patients were mainly grade 1–2, in KEYNOTE-010 (Herbst et al., 2016), two cases (less than 1%) were reported as grade 3–4 rash, with a median time of AEs of 5–7 weeks.

In the study of malignant melanoma, CheckMate 066 (Robert et al., 2015a) reported one case of grade 3–4 rash and one case of grade 3–4 pruritus. The incidence of grade 3–4 cutaneous AEs was 0.5%. CheckMate 037 and KEYNOTE-002 did not report grade 3–4 cutaneous AEs (Ribas et al., 2015; Weber et al., 2015). The incidence of Nivolumab and Pembrolizumab associated rashes was 14.3 and 16.7% respectively, and the incidence of rash over grade 3 was 1.2 and 1.9% respectively, according to the meta-analysis of V. R. Belum (Belum et al., 2016). Skin reactions generally occur two to 3 weeks after the administration of the immune inhibitor (Kumar et al., 2017). Studies have shown that progression‐free survival and overall survival in cancer patients with skin reactions are longer than those without skin reactions, after immunotherapy (Sanlorenzo et al., 2015; Hasan Ali et al., 2016; Imafuku et al., 2017; Min Lee et al., 2018; Quach et al., 2019). A similar conclusion was reported in a study of immunotherapy combined with radiotherapy (Haratani et al., 2018). Quach HT (Quach et al., 2019) retrospective analysis of 318 patients treated with anti-PD-1 monoclonal showed that patients who experienced skin toxicity 3 months after the application of the immune inhibitors, had better therapeutic outcomes than those who did not show skin issues, and that a higher response rate was observed in patients with vitiligo and pruritus than patients with only vitiligo.

Clinical manifestations of severe bullous skin reaction caused by PD-1 monoclonal antibody are very similar to those observed in SJS/TEN. However, the former characterizes by the duration of drug application, the involvement of the mucous membrane, and the degree of skin peeling (Lipowicz et al., 2013; Zhao et al., 2018). Research by Robin Reschke et al. (2019) showed that among all the current reports of serious skin reactions caused by checkpoint inhibitor therapy, only a small number are typical SJS/TEN. Therefore, clinicians, especially oncologists, should differentiate them by considering clinical evidence and if necessary, request a dermatologist assistance in diagnosis and treatment.

### Case Reports on Toxic Epidermal Necrolysis Induced by Immunotherapy

Although mild dermatotoxicity indicates a better clinical outcome, severe dermatotoxicity may result in treatment interruption (Macdonald et al., 2015), and sometimes it may become a life-threatening condition (Nayar et al., 2016; Vivar et al., 2017; Logan et al., 2020). TEN is the most serious type of drug eruption, characterized by extensive epidermal exfoliation and blistering (Roujeau and Stern, 1994), with a mortality of 25–50% (Mockenhaupt et al., 2008; Kinoshita and Saeki, 2017).

Three cases of TEN induced by anti-PD-1 in melanoma have been reported to date (Nayar et al., 2016; Vivar et al., 2017; Logan et al., 2020), all of which were treated with Nivolumab, and a previous history of Ipilimumab or were treated with these drugs in combination. TEN developed after the second or third cycle of Nivolumab. In all cases, TEN was treated with immunoglobulins (IVIg), two patients used cyclosporines, and other two used glucocorticoids. All patients finally died, including those reported in this article (Table 1 **).**

**TABLE 1:** Reported cases of TEN caused by anti-PD-1 monoclonal antibodies therapy.

	Publication year	Authors	Patient age/sex	Cancer type	Immunotherapy	History of immunotherapy	TEN time	Treatment for TEN	Outcome
1	2016	Namrata Nayar et al. (2016), Weber et al. (2015)	64/F	Melanoma	Nivolumab (3 mg every 2 weeks)	Ipilimumab (3 mg/kg for one cycle)	After 2nd cycle	Prednisone, methylprednisolone cyclosporine, IVIg	Died because of disease progression and sepsis
2	2017	Vivar et al. (2017), Yang et al. (2019)	50/F	Melanoma	Nivolumab (1 mg/kg for 1st cycle,3 mg/kg for additional two)	Ipilimumab (3 mg/kg for one cycle)	After 3rd cycle	Prednisone, infliximab, IVIg	Died because of sepsis and multisystem organ failure
3	2020	Logan et al. (2020), Nayar et al. (2016)	62/M	Melanoma	Ipilimumab (3 mg/kg) +Nivolumab (1 mg/kg)	None	After 2nd cycle	IVIg, Cyclosporine, G-CSF	Died (the author did not mention the reason)
4		This article	38/F	Cervical cancer	Sintilimab (200 mg every 3 weeks for 1st 2nd and 4th cycle) Toripalimab (3 mg/kg for 3rd cycle)	None	After 4th cycle	Methylprednisolone, IVIg	Died from septic

The incidence of CTLA-4 adverse reactions is as high as 60%, and skin and gastrointestinal tract is the most easily affected organs (Hodi et al., 2010). The incidence of grade 3–4 adverse events was 20% (Schachter et al., 2017). At present, it is believed that the cause of CTLA-4 adverse reactions was the excessive immune response to normal organs after activation of T cells (Blansfield et al., 2005). However, the mechanism that causes TEN is still unclear.

### Relationship Between Anti-Programmed Death-1 Monoclonal Antibodies and Cutaneous Reactions

Immune-related AEs may damage any organ in the body (Michot et al., 2016), mostly skin (Minkis et al., 2013; Abdel-Rahman et al., 2015; Naidoo et al., 2015). However, the mechanism of anti-PD-1-induced skin toxicity has not yet been elucidated (Maloney et al., 2020). PD-1/PD-L1 pathway plays an important role in autoimmunity, preventing T cells from responding to autoantigens (Francisco et al., 2010). After anti-PD-1 monoclonal antibody administration, this balance may be broken, causing T cells to attack normal and tumor cells, leading to toxic and side effects (Berman et al., 2015). In the PD-1 knockout mouse model, Okazaki et al. (2003) demonstrated that PD-1 blockade not only affected Treg function, but also participated in the production of autoantibodies. Similar results were observed in melanoma patients treated with Nivolumab (Kanameishi et al., 2016). The three reported cases of TEN induced by anti-PD-1 had a medical history of anti-CTLA-4. Gu et al. (2019) meta-analysis showed that anti-PD-1/PD-L1 combined with anti-CTLA-4 caused a higher incidence of adverse reactions and was prone to treatment discontinuation, as shown in Check-Mate 067 study (Larkin et al., 2015). However, results evidenced higher remission rates when two immune inhibitors were used in combination (Robert et al., 2015b). Choi et al. (2019) reported a case of liver cancer patient who successfully switched to pembrolizumab due to nivolumab allergy. Therefore, different anti-PD-1 monoclonal antibodies share the same mechanism, but may cause different reactions. Our patients presented with TEN after using two different anti-PD-1 monoclonal antibodies. However, interaction between these two drugs may not be discarded.

### Treatment of Skin Reactions Caused by Immunotherapy

Early treatment of rash caused by immune agents is particularly important (O’Kane et al., 2017). In general, patients can be topically treated with glucocorticoid ointment and orally with antipruritics (mainly antihistamines) if the patient presents itching. For grade 3–4 toxic side effects caused by anti-PD-1 monoclonal antibodies, glucocorticoids should be orally administered and anti-PD-1 should be discontinued until glucocorticoid dose reduction therapy is completed (Eigentler et al., 2016; Weber et al., 2017). The drug should be immediately discontinued and the patient should be hospitalized if TEN develops. SJS/TEN treatment guidelines from the United Kingdom (Creamer et al., 2016) and the literature review of Friedman et al. (2016) indicate that glucocorticoids, immunoglobulins, and cyclosporine may be used in TEN therapy but such treatment has not been clearly demonstrated over supportive therapy alone. Previous studies (de Sica-Chapman et al., 2010) have shown that G-CSF has immunomodulatory effects and promotes epithelial regeneration. SJS/TEN treatment by plasmapheresis has also been reported (Narita et al., 2011). In this regard, Koštál used plasmapheresis therapy in four patients presenting TEN who did not respond to glucocorticoids and immunoglobulin. Their symptoms improved and necrotic epithelium began to repair (Kostal et al., 2012). Skin care played an important role in the recovery from TEN (Cooper, 2012).

## Conclusion

Although most skin reactions caused by anti-PD-1 monoclonal antibodies were mild, they may still cause fatal skin toxicity. Therefore, early recognition, intervention, and treatment are essential to avoid further development of skin reactions. In this regard, early application of glucocorticoids may slow the response.

## Data Availability

The original contributions presented in the study are included in the article/Supplementary Material, further inquiries can be directed to the corresponding author.

## Abbreviations

AEs, adverse effects; BSA, body surface area; PD-1, Anti-programmed cell death protein 1; TEN, toxic epidermal necrolysis.
